# Metacarpal Index Estimated by Digital X-ray Radiogrammetry as a Tool for Differentiating Rheumatoid Arthritis Related Periarticular Osteopenia

**Published:** 2006-09

**Authors:** Joachim Böttcher, Alexander Pfeil, Alexander Petrovitch, Mirco Schmidt, Anika Kramer, Max Ludwig Schäfer, Mieczyslaw Gajda, Gert Hein, Gunter Wolf, Werner A. Kaiser

**Affiliations:** 1 *Institute of Diagnostic and Interventional Radiology, Friedrich Schiller University Jena Erlanger Allee, Jena, Germany;*; 2 *Department of Pathology, Friedrich Schiller University Jena, Bachstrasse, Jena, Germany;*; 3 *Clinic of Internal Medicine III, Department of Rheumatology and Osteology, Friedrich Schiller University Jena, Erlanger Allee, Jena, Germany*

**Keywords:** digital X-ray radiogrammetry, dual energy X-ray absorptiometry, metacarpal index, bone mineral density, rheumatoid arthritis, steinbrocker stage

## Abstract

To investigate Metacarpal Index (MCI) and Bone Mineral Density (BMD) estimated by Digital X-ray Radiogrammetry (DXR) with respect to its ability to quantify severity-dependent variations of bone mineralisation in patients with early rheumatoid arthritis compared to Dual Energy X-ray Absorptiometry (DXA), 122 patients underwent a prospective analysis of BMD and MCI by DXR, whereas both DXR-parameters were estimated from plain radiographs of the non-dominant hand. In comparison DXA measured BMD on total femur and lumbar spine (L2-L4). Additionally Steinbrocker Stage was assessed to differentiate the severity of rheumatoid arthritis (RA). Disease activity of RA was estimated by C-reactive Protein (CRP; in mg/l), Erythrocyte Sedimentation Rate (ESR in mm/1st hour) and by the disease activity score with 28-joint count (DAS 28). In consequence, The DXR-parameters, in particular DXR-MCI, revealed significant associations to age, Body Mass Index, CRP, DAS 28 and Steinbrocker graduation; no significant associations could be verified between DXA-parameters and all characteristics of disease activity and severity of RA. The highest correlation was found between DXR-MCI and DXR-BMD with R=0.89 (independent from severity of RA). In all patients DXR-MCI significantly decreased (-14.3%) from 0.42 ± 0.09 (stage 1) to 0.36 ± 0.07 (stage 2) dependent on severity of RA. The comparable relative reduction of DXR-BMD was -11.1%. The group of patients with minor disease activity (DAS 28>5.1) showed a significant flattened reduction (-11.4%) for DXR-MCI from 0.44 ± 0.08 (stage 1) to 0.39 ± 0.08 (stage 2). For accentuated disease activity (DAS 28>5.1) the DXR-MCI revealed a pronounced reduction (-23.1 %). No significant declines were observed for DXA-BMD of the lumbar spine and total femur in all patients as well as dependent on disease activity. Conclusion: DXR can exactly quantify cortical thinning of the metacarpal bones and can identify cortical demineralisation in patients suffering from early rheumatoid arthritis surpassing DXA-measurements at axial bone sites. In this context DXR-MCI seems to be the most sensitive parameter for differentiation of patients with minor or accentuated disease activity following severity-dependent cortical bone loss.

## INTRODUCTION

Rheumatoid arthritis (RA) is a systemic inflammatory disease; in 80% of patients with RA the small joints of the hand are affected leading to destruction of periarticular tissue, including juxta-articular bone (1). Osteoporosis is a major clinical complication in RA and occurs in two forms: periarticular osteopenia in near proximity to inflamed joints, which is a typical phenomenon in early RA, and generalised osteoporosis affecting the axial and appendicular bones occurring during the course of rheumatoid disease (2-4). Generalised bone loss may be influenced by immobility, the inflammatory process itself and treatments such as steroids, whilst periarticular demineralisation is probably caused by local release of inflammatory agents (5). Many studies have revealed the influence of different cytokines with respect to the dysregulation of bone and cartilage remodelling (6). Recently, receptor activators of nuclear κB ligand (RANKL) and osteoprotegerin (OPG), a decoy receptor for RANKL, have been identified as central regulators of osteoclast activation and differentiation. OPG and RANKL production is modulated by various cytokines, growth factors and hormones. In affected synovium both fibroblasts and activated T cells express RANKL and maintain osteoclast activation and differentiation. Thus, OPG and RANKL are important molecular agents which appear to systemically influence bone resorption in the juxta-articular bone (7).

Osteoporosis in RA, which results in low bone mineral density and microarchitectural deterioration of bone tissue and leads to diminished biomechanical competence of the skeleton, commonly leads to low-trauma or atraumatic fractures, particularly at the spine, hip and wrist. Periarticular demineralisation is also the first disease-related morphological sign before erosions and joint space narrowing occur (8). Therefore various osteodensitometric techniques are concentrated on the quantification of RA-related bone loss, but the early detection of periarticular demineralisation is further on unsatisfactory.

Digital X-ray Radiogrammetry (DXR) seems to bridge this gap in patients with rheumatoid arthritis and also generalised osteoporosis as a new diagnostic approach using digitised radiographs (9-13). DXR is based on conventional radiogrammetry, which is a radiogeometric technique used to estimate the thickness of the cortical bone partition. For over 40 years conventional radiogrammetry has been the primary method for assessment of bone status from hand radiographs (14-16). This conventional method measures the total width of a bone and the medullary width at the mid-point of the second metacarpal of the non-dominant hand. The measured data are methodically used to calculate various indices such as the Metacarpal Index (ratio of total bone width and cortical thickness), the Barnett and Nordin Index (percentage cortical thickness) (14), the Garn Index (cortical area) (17) and the Exton-Smith index (related to cortical area and surface area) (18) and thus to quantify changes of cortical bone in case of osteoporosis. Conventional radiogrammetry and the above-mentioned indices are most sensitive to cortical bone changes, i.e. periosteal apposition and especially endosteal resorption. Despite conventional radiogrammetry being relatively inexpensive and widely available, clinical applicability is limited because of imprecision due to the difficulty in identification of the endosteal margin and the precise marking of the mid shaft location by the operator-dependent radiogrammetrical measurements.

Adams *et al*. (19) showed data of intra- and inter-observer reproducibility with coefficients of variation between 8% and 11%. Various improvements to the initial radiogrammetric method were suggested, including averaging over multiple measurement sites on the same bone (20) and averaging over several bones. Aguado *et al*. (21) used digitally enlarged radiographs to improve the visual resolution resulting in coefficients of variation up to 2.4%.

The availability of digital images have improved the applicability of radiogrammetry for quantitative measurements of radiogeometrical features (13).

Digital X-ray Radiogrammetry is a new operator-independent diagnostic tool, providing automated measurements of cortical bone mineral density (DXR-BMD) and Metacarpal Index (DXR-MCI) on the metacarpals using digitised radiographs. Böttcher *et al*. (22) observed an excellent intra- (0.05-0.33%) and inter-observer reproducibility (0.26-1.54%) for DXR. Furthermore this study revealed that most of the image-capturing conditions (i.e. film-focus-distance, film sensitivity, film brand, exposure level) did not significantly affect the DXR-measurements and therefore DXR has provided estimates of cortical bone partition with high precision and remarkable reproducibility (23). The high precision of DXR is caused by refinement, computerisation and the implementation of algorithms for automatic and operator-independent image analysis, which is based on a combined computerised radiogrammetric and textural analysis of the three middle metacarpal bones (24).

Although the DXR-estimates are not taken at a point known for a high incidence of fracture, in a prospective study Bouxsein *et al*. (10) documented that DXR showed an equal prediction value of fracture risk compared to Single Photon Absorptiometry regarding fractures of the wrist, spine and femur. Moreover Hyldstrup *et al*. (11) verified an increase of DXR-BMD and DXR-MCI in post-menopausal women after treatment with bisphosphonates, whereas no improvement of bone partition could be documented by Dual Energy X-ray Absorptiometry (DXA)-measurements of the spine, femur and forearm. In addition post-menopausal women using hormone replacement therapy showed a significant increase of MCI calculated by DXR. Rosholm *et al*. (25) revealed a significant association between DXR- and DXA-parameters with R=0.86 (distal radius), R=0.73 (femur) and R=0.62 (lumbar spine) in healthy women. Additionally Ward *et al*. (13) found close correlations between DXR and DXA at the axial measurement site versus Single Energy X-ray Absorptiometry measured at the forearm.

The results of another study (9) demonstrated a better sensitivity for identifying women both with and without osteoporosis (e.g. positive and negative predictive value) by DXR compared to quantitative ultrasound, and suggested that DXR-BMD and phalangeal radiographic absorptiometry might be most effective to identify high-risk postmenopausal women.

Another promising application of DXR-technology is the determination of peripheral bone status in childhood. Children with cystic fibrosis, Turner syndrome, Marfan syndrome, osteogenesis imperfecta, inflammatory bowel diseases and renal disorders often develop disease-related or therapy-induced osteopenia, frequently requiring radiographs of the hand for disease monitoring based on bone age determination, which could be used for DXR-estimates. Mentzel *et al*. (26) published that DXR-BMD seemed to be a reliable technique in children for quantification of cortical bone loss following renal transplantation. Additionally a study presented by van Rijn *et al*. (27) found significant correlations between DXR and DXA measured at lumbar spine and total body in lymphoblastic leukaemia and revealed an increase of DXR-BMD during treatment of children with growth hormone deficiency.

The most important application of DXR is the quantification of periarticular bone loss in rheumatoid arthritis. In comparison to other osteodensitometric techniques (DXA, peripheral Quantitative Computertomographie, Quantitative Ultrasound), DXR can detect and quantify the disease-related cortical bone loss caused by the course and severity of rheumatoid arthritis (28-31).

This cross-sectional study evaluates the ability of performing a precise quantification of the radiogeometrical detectable periarticular demineralisation by DXR dependent on severity of RA, differentiated for disease activity and compared to Dual Energy X-ray Absorptiometry.

## PATIENTS AND METHODS

### Patients

122 patients (100 female/22 male) were selected in a prospective study from a data pool of patients treated in our outpatient clinic. Mean age was 57.2 years with a standard deviation of 13.0 years and an age range of 18 to 80 years. All patients showed a disease duration of less than two years to include only patients with early RA in this trial.

Inclusion criteria for the study were a clinical proven existence of rheumatoid arthritis following the revised ACR-criteria from 1987 (32), the availability of digitally performed radiographs of the non-dominant hand (using standardised technical parameters) as well as the measurement of DXA-BMD on total femur and lumbar spine within two weeks.

Thirty-one patients were treated with methotrexate and 53 subjects with other disease-modifying anti-rheumatic drugs (i.e. DMARDs) in combination with nonsteroidal anti-inflammatory drugs (i.e. NSAIDs). 34 patients were on long-term low-dose prednisolone therapy (5 mg per day over a 6 month period). The remaining four patients had neither received systemic corticosteroids and DMARDs nor immune modulating drugs. Subjects with abnormal renal function (serum creatinine >130 μmol/l), or on hormone replacement therapy/biphosphonates or having other conditions known to affect bone metabolism were excluded. Further exclusion criteria were signs of fracture and visible osteosynthetic material regarding the right and left upper extremities (including clavicle, humerus, ulna, radius and hand).

A preselection regarding severity of rheumatoid arthritis was performed using the Steinbrocker graduation, which considered only the most affected hand joint (for definition see reference 33, 34 and also 28). Each X-ray was independently scored by two trained observers.

In cases of ambiguity, a third radiologist reviewed the radiographs; the individual sum of scoring points was then divided by the evaluated joints and the patients were subdivided into the different groups of grading according to the Steinbrocker Stages (Stage 1: n=32; Stage 2: n=90).

The process of disease activity was characterised by the concentrations of C-reactive Protein (CRP) and the erythrocyte sedimentation rate (ESR). CRP was measured by nephelometric assay to estimate disease activity of RA. To consider clinical characteristics the Disease Activity Score with 28-joint count (DAS 28) was additionally provided. DAS 28 as a very sensitive parameter to estimate disease activity in RA was used to differentiate between patients with minor (DAS 28≤5.1; n=72) and accentuated disease activity (DAS 28>5.1; n=50), which has been revised to include a simplified, ungraded 28-joint count for tenderness (tender joint count; TJC) and for swelling (swollen joint count; SJC). The DAS 28 is calculated as follows (35):

DAS 28 = (0.56 × TJC^½^) + (0.28 × SJC^½^) + (0.7 × ln [ESR]) + (0.014 × GH).

GH designates general health, as assessed by the patient using a 100mm visual-analog scale (“How do you feel concerning your arthritis?” 0=very well and 100=extremely bad);

ln ESR is the natural logarithm of the Erythrocyte Sedimentation Rate (ESR in mm/1^st^ hour; determined by the Westergreen method).

### Methods

**Dual Energy X-ray Absorptiometry (DXA).** Bone mineral density and bone mineral content of the lumbar vertebrae (L1-L4) and the left total femur (sum of femoral neck, trochanter, intertrochanteric regions) were measured by DXA (Hologic QDR 4500, Hologic Inc., Bedford, Mass., USA). Short-term precision of the DXA equipment was CV=1%.

**Digital X-ray Radiogrammetry.** The Pronosco X-Posure System™ (Version 2.0, Sectra Pronosco A/S, Vedbaek, Denmark), which involved a radiogrammetric and textural analysis of the three middle metacarpal bones, was used to determine BMD and MCI based on Digital X-ray Radiogrammetry, requiring radiographs of the non-dominant hand. All plain radiographs were acquired by a Polydoros SX 80 (Siemens, Munich, Germany) under the following standardised conditions: Filter with 1.0 mm thickness related to aluminium 80, tube voltage 42 kV, exposure level 4 mAs, film focus distance 100 cm, Scopix Laser 2 B 400 (Agfa GmbH and Cie. KG, Cologne, Germany). The digital radiographs were printed and subsequently scanned into the system at a resolution of 300 dpi, corresponding to 118 pixels per centimetre. The system itself checked the quality of the scanned images and interrupted the examination in case of inadequate quality. The computer algorithms automatically defined regions of interest (ROIs) around the narrowest bone parts of the metacarpalia II, III and IV and subsequently specified the outer and inner cortical edges of the studied cortical bone parts. To locate the bones in the radiograph, the Pronosco X-posure System™ applied a model-based algorithm known as the Active Shape Model (ASM). The ASM algorithm was adapted to find the diaphysis of the three middle metacarpals in the hand (25). After each diaphysis was identified, regions of interest (ROI) are placed automatically for each metacarpal.

In detail the algorithm placed the three ROIs in a coupled fashion by sliding them in a partly fixed configuration along the bone shafts to a position identified by the minimum combined bone width. The heights of the ROIs were fixed to 2.0 cm, 1.8 cm, and 1.6 cm for the 2nd, 3rd, and 4th metacarpal, respectively.

Within each ROI, the endosteal (inner) and periosteal (outer) edges were automatically found, thereby segmenting the bone into two cortical regions and one endosteal region. Along a profile across the bone, the two endosteal edge points were associated with the points of maximal intensity. The periosteal edge points were characterised with the points of maximal curvature, which in this case was similar to the gradient.

Apart from placing the radiography on the CCD-based desktop flatbed scanner, there was otherwise no operator interaction connected to the DXR measurement. The analysed images and their ROIs were displayed on the computer monitor. The mean of the cortical thickness (MCP-CT_mean_) and overall bone cortical thickness (CT) of the second, third and fourth metacarpal were estimated as given by Rosholm *et al*. (25):

(MCP-CT_2_ + MCP-CT_3_ + MCP-CT_4_)/3 = MCP- CT_mean_.

**Figure 1:**
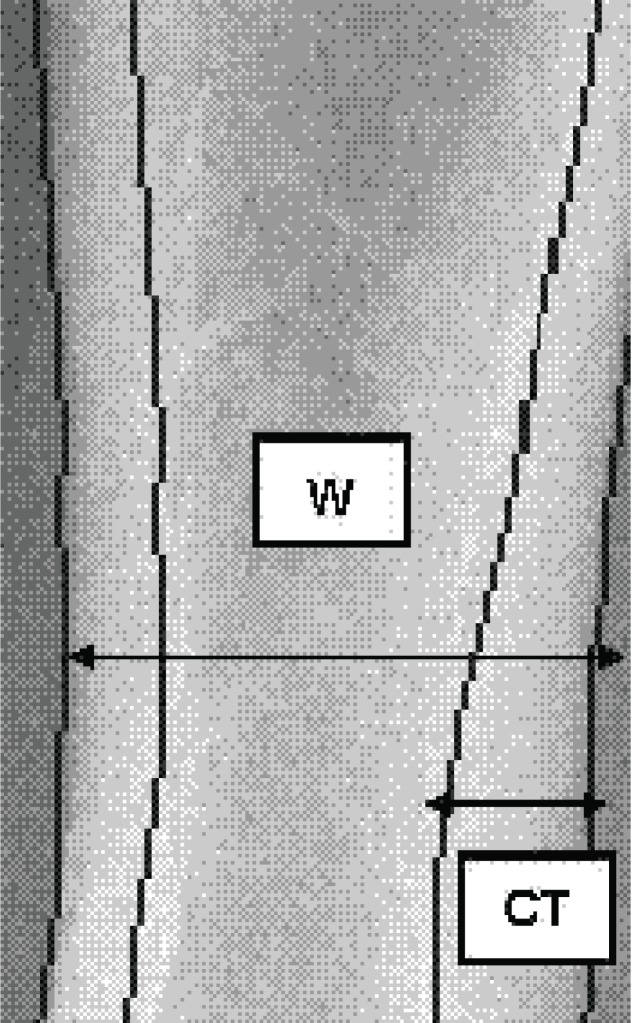
Principle for calculation of Metacarpal Index (MCI) by Digital X-ray Radiogrammetry. W, outer bone width of a metacarpal; CT, cortical thickness.

Subsequently, the cortical volume per area (VPA, cm) was computed for each metacarpal assuming a cylindrically-shaped bone based on cortical thickness (CT) and mean outer bone diameter (W) as given by Lazenby (36) and Rosholm *et al*. (25; see Figure 1):

VPA = π × CT × (1 - CT/W).

The total VPA for the metacarpals was defined as a weighted average:

VPA_mc_ = (VPA_2_ + VPA_3_ + 0.5 VPA_4_) / 2.5.

The fourth metacarpal was given a lower weight due to a lower precision and an inferior clinical importance.

The final DXR-BMD -based on the mean VPA- was then calculated with a below-mentioned correction for the estimated porosity index (P):

DXR - BMD = c × VPA × (1 - P).

The scaling constant c was determined so that DXR-BMD on the average was equal to that of the mid-distal forearm region of the Hologic QDR 2000 densitometer (Hologic Inc., Bedford, Mass., USA). The constant adapted VPA to both the volumetric mineral density of compact bone and the typical shape characteristics of the involved bones.

The porosity index was a technical parameter given as a value between 1 and 19 which was derived from the area percentage of local intensity minima found in the cortical part of the bone relative to the entire cortical area (25, 37). Consequently the porosity index represented an estimated three-dimensional cortical porosity, aimed to be the fraction of the cortical bone volume that was not occupied by bone.

The metacarpal index (DXR-MCI) obtained the mean cortical thickness (CT) normalised with the mean outer bone diameter (W) for each bone part as follows (24, 25; see Figure 1):

DXR-MCI = 2 × CT / W.

### Short-term precision of Digital X-ray Radiogrammetry

**Inter-radiograph reproducibility** (measurement by acquiring ten repeated radiographs of the same hand with re-positioning under standard X-ray settings; 22): DXR-MCI: 0.24% DXR-BMD: 0.31%.

**Intra-radiograph reproducibility** (measuring one identical image of the same hand for 10 times; 22): DXR-MCI: 0.14% DXR-BMD: 0.09%.

### Ethics

All examinations were performed in accordance with the rules and regulations of the local human research and ethics committee. As special note the authors emphasise that all radiographs used for DXR-calculations and all DXA-measurements were routinely performed, without exception based on clinical considerations. No patient additionally underwent both DXA-measurements and the performance of X-ray imaging (for DXR calculation) because of research purposes.

### Statistics

Results were expressed as mean and standard deviation (SD). The normality of the data was checked using the Kolmogorov-Smirnov test. The associations among the investigated variables were examined by linear regression analysis. The significance of severity-dependent bone loss were calculated with the Mann Whitney U test. Significance was set at *p*<0.05. Statistical analysis was performed using SPSS version 10.13 (SPSS, Chicago, USA), for Windows.

## RESULTS

### Comparison between DXR-versus DXA-para-meters and the demographic, anthropometric and clinical characteristics (Table 1)

Measurements of DXA and DXR were performed in all patients. All correlations between Steinbrocker Stage and the different parameters of DXR were significant, whereas DXR-MCI (R=–0.40, *p*<0.01) showed a closer association compared with DXR-BMD (R=–0.34, *p*<0.05). However, for DXA-BMD measured on lumbar spine and total femur no significant correlation was observed.

**Table 1:** Associations between demographic, anthropometric and clinical characteristics and DXA- versus DXR-parameters

n=122	DXA-BMD (total femur)	DXA-BMD (lumbar spine)	DXR-BMD	DXR-MCI

Age	R=-0.44^b^	R=-0.41^b^	R=-0.54^a^	R=-0.61^a^
BMI	R=0.13*	R=0.09*	R=0.42^b^	R=0.37^b^
Swollen joint	R=-0.12*	R=-0.06*	R=-0.15*	R=-0.19*
tender joint	R=-0.25^c^	R=-0.27^c^	R=-0.09*	R=-0.03*
CRP	R=-0.16*	R=-0.19*	R=-0.32^c^	R=-0.31^c^
Steinbrocker Stage	R=-0.15*	R=-0.09*	R=-0.34^c^	R=-0.40^b^
ESR	R=-0.02*	R=-0.08*	R=-0.13*	R=-0.11*
DAS 28	R=-0.20*	R=-0.18*	R=-0.45^b^	R=-0.52^a^

* Not significant;

a *P*<0.001;

b *P*<0.01;

c *P*<0.05;

BMD, Bone Mineral Density; BMI, Body Mass Index; DAS, Disease Activity Score; DXA, Dual Energy X-ray Absorptiometry; DXR, Digital X-ray Radiogrammetry; CRP, C-reactive Protein; ESR, Erythrocyte Sedimentation Rate; MCI, Metacarpal Index.

A significant negative association was shown between the DXR-MCI (R=-0.31, *p*<0.05) as well as DXR-BMD (R=-0.32, *p*<0.05) and C-reactive Protein, but also not for DXA-data. No significant associations could be documented for Erythrocyte Sedimentation Rate and the different parameters of DXR and DXA. Otherwise significant associations were observed for DAS 28 and DXR-para-meters, whilst DXA-parameters again failed the significant level. Additionally DXA-parameters demonstrated significant correlations for age and tender joints, DXR-data for age and Body Mass Index.

Severity-dependent demineralisation using graduation from Steinbrocker (Table 2): For all patients DXR-BMD and DXR-MCI were significantly correlated to DXA-BMD of lumbar spine and total femur. The closest association, which compared both techniques, was observed between DXR-BMD and DXA-BMD of the total femur (R=0.59, *p*<0.01). Coefficients of correlation between the different DXR- and DXA-parameters of Steinbrocker Stage 1 were significantly higher compared to Steinbrocker Stage 2.

**Table 2:** Reduction of bone mineral density from stage 1 to 2 (Steinbrocker Stage) dependent on severity of rheumatoid arthritis in all patients

n = 122	Steinbrocker Stage 1 mean (SD) n=32	Steinbrocker Stage 2 mean (SD) n=90	Relative difference from stage 1 to stage 2

DXR-BMD in g/cm^2^	0.51 (0.09)	0.45 (0.08)	-11.1%^b^
DXR-MCI	0.42 (0.09)	0.36 (0.07)	-14.3%^a^
DXA-BMD in g/cm^2^ (total femur)	0.90 (0.14)	0.88 (0.16)	-2.2%*
DXA-BMD in g/cm^2^ (lumbar spine)	0.87 (0.14)	0.85 (0.15)	-1.9%*

* Not significant;

a *P*<0.001;

b *P*< 0.01.

DXA, Dual Energy X-ray Absorptiometry; DXR, Digital X-ray Radiogrammetry; BMD, Bone Mineral Density; MCI, Metacarpal Index; SD, Standard Deviation.

Close correlations were found for DXR-MCI versus DXR-BMD with R=0.89 (*p*<0.001, Steinbrocker Stage 1) as well as R=0.88 (*p*<0.001, Steinbrocker Stage 2) and for DXA-BMD of lumbar spine versus DXA-BMD of the total femur with R=0.70 (*p*<0.001, Steinbrocker Stage 1) as well as R=0.63 (*p*<0.001, Steinbrocker Stage 2).

Closer associations could be observed between DXA-BMD of the distal radius and DXR-BMD (R=0.79; *p*<0.001) versus DXR-MCI (R=0.65; *p*<0.001) in a limited group (n=21) of our study cohort, pointing at the fact of a better discrimination regarding periarticular osteopenia by DXA-measurements at the distal radius.

For Steinbrocker Stage DXR-MCI significantly decreased (-14.3%) from 0.42 ± 0.09 (stage 1) to 0.36 ± 0.07 (stage 2) in the total study cohort. The relative reduction of DXR-BMD was -11.1%. Otherwise DXA-BMD of the total femur and also of the lumbar spine showed a non-significant reduction up to -2.2%.

### Influence of disease activity

**Figure 2:**
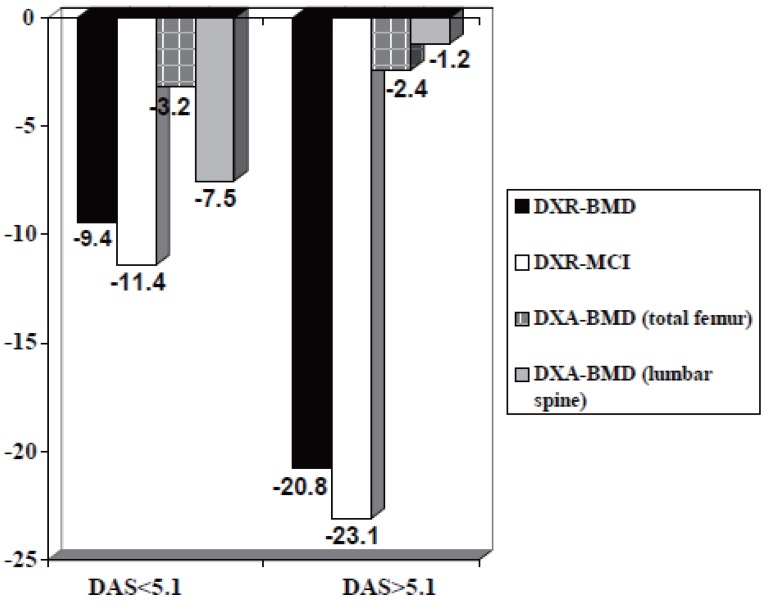
Relative reduction of DXR- and DXA-parameters from stages 1 to stage 2 (Steinbrocker graduation) differentiated for disease activity of rheumatoid arthritis based on DAS 28 (Disease Activity Score 28-joint count).

**Minor activity of rheumatoid arthritis (Figure 2):** DXR-MCI revealed a significant reduction (-11.4%) from 0.44 ± 0.08 (stage 1) to 0.39 ± 0.08 (stage 2) and DXR-BMD significantly decreased (-9.4%) from 0.53 g/cm^2^ ± 0.07 (stage 1) to 0.48 g/cm^2^ ± 0.10 (stage 2). A non-significant decline of -7.5% for DXA-BMD (lumbar spine) from 0.93 g/cm^2^ ± 0.12 (stage 1) to 0.86 g/cm^2^ ± 0.15 (stage 2) was observed. DXA-BMD (femur) showed no significant change between stage 1 and 2 (-3.2%).

**Accentuated activity of rheumatoid arthritis (Figure 2):** The DXR-BMD showed a significant decline (-20.8%) from 0.48 g/cm^2^ ± 0.04 (stage 1) to 0.38 g/cm^2^ ± 0.07 (stage 2) and DXR-MCI also revealed a reduction (-23.1%) from 0.39 ± 0.04 (stage 1) to 0.30 ± 0.05 (stage 2).

DXA-BMD (lumbar spine) decreased from 0.84 g/cm^2^ ± 0.11 (stage 1) to 0.83 g/cm^2^ ± 0.16 (stage 2) with a relative reduction of -1.2%. Regarding DXA-BMD (total femur) a reduction of -2.4% from 0.84 g/cm2 ± 0.10 (stage 1) to 0.82 g/cm^2^ ± 0.12 (stage 2) was documented.

## DISCUSSIONS

Most patients with early rheumatoid arthritis are characterised by apparently normal hand radiographs despite severe immune and clinical involvement of the small hand joints and then show destructive joint alterations within the first two years after onset of RA. The metacarpal osteopenia predates periarticular erosions and joint destruction (31). Therefore the early quantification of periarticular RA-related demineralisation implies an important prognostic benefit for the patients. Osteoporosis occurs more frequently in patients with RA than in healthy individuals and represents the earliest radiological characteristic of RA (8). Recent studies verified the coexistence of periarticular and generalised demineralisation in RA (38). Periarticular osteoporosis has been shown to have a close association to level of disease activity, but not to disease duration, even indicating a maximal demineralisation in early RA (39-41). Kalla *et al*. (42) also verified a significant reducing effect on metacarpal osteopenia in patients on antirheumatic therapy.

This study reveals a significant severity-related reduction of periarticular cortical bone mineral density and in particular regarding the Metacarpal Index using DXR, comparable to recently published results (43). Additionally, Stewart *et al*. (31) reported that DXR is a reliable predictor for the erosive manifestation of RA. The authors found that the reduction of DXR-BMD after 1 year was very specific (100%) and the technique was highly sensitive (63%) in detecting patients who developed an accelerated progress of RA with occurrence of erosions after a 4 year observation period.

Böttcher *et al*. (28) revealed a marked severity-dependent DXR-MCI and DXR-BMD decline for patients in different stages of RA, and no significant loss of BMD as estimated by DXA of the total femur and lumbar spine. Furthermore the data of this study demonstrate the close association between DXR-parameters and parameters of disease activity as well as severity of RA surpassing DXA-estimates, which could also be confirmed by Jawaid *et al*. (34).

In this context DAS 28 and CRP seems to better describe disease activity compared to ESR which shows no significant correlation to DXR- and DXA-data. Whereas the association between DXR-BMD and DXR-MCI reveals no change in Steinbrocker Stage 1 and 2, the associations regarding DXA-data are diminished in stage 2 pointing at the declined detection of periarticular osteopenia by DXA. Altogether DXR can better discriminate the RA-related periarticular cortical bone loss with consideration of disease activity and severity compared to DXA, whereas DXR-MCI is the most sensitive parameter surpassing DXR-BMD. Potentially the DXA-measurements at the distal radius can be more appropriate to discriminate periarticular RA-related osteopenia based on the fact of a closer association of DXR-parameters and DXA-data estimated at peripheral (i.e. radius) bone site.

DXR is ideal for the quantification of periarticular demineralisation without an interference caused by soft tissue (44), because this technique utilises the metacarpals as the measurement site. Because of a frequent and severe involvement of metacarpal joints in the rheumatoid inflammatory process, Alenfeld *et al*. (41) observed a higher degree of bone loss in the subregions of phalanges and metacarpals in comparison with whole-hand BMD reduction. The influence of disease-related bony defects and erosions in the DXR calculations can be minimised because of DXR measurements on the diaphyseal part of the metacarpal bones.

In addition the short-term precision of DXR at approximately 0.40 % is at a very low level (22), indicating that estimated demineralisation is in fact disease-related and not based on the precision error of the osteodensitometric method itself.

Beside the operator-independent function of DXR, the high precision of DXR may also be explained by the procedure to detect the inner cortical edges of a bone in particular for patients with RA-related cortical thinning (25). The detection of the inner cortical edge is responsible for the precise calculation of the metacarpal cortical thickness and, consequently, the DXR-BMD estimate.

In the image analysis of DXR, the inner cortical edge is associated with the intensity maximum of the intensity profile across the bone of interest.

For a bone with thick cortical tissue, the intensity maximum is located on a broader and less curved top than for a bone with a thin cortical shell. This property implies that the position of the intensity maximum is more precisely defined on the narrow top of a thin bone than on the broader top of a thick bone (24).

A possible limitation of DXR may be the measurement of only the cortical partition of BMD, because the cortical bone matrix implies minor bone metabolism compared to trabecular bone tissue. Otherwise cortical thinning of periarticular bone, enhanced by the inflammation process, is a typical phenomenon of bone destruction in rheumatoid arthritis (42), which can be assumed because of very high bone turnover on the inner and also outer bone surface (45). It is also well known that osteoporosis in postmenopausal women is characterised by both the reduction of cortical thickness and a decrease of trabecular bone volume (46, 47).

Furthermore the influence of body constitution on the cortical bone partition could be also documented in another DXR-study (44), which revealed an increase of DXR-MCI and DXR-BMD in individuals with advanced Body Mass Index. In addition normative reference data for DXR are now available (48), which guarantee a better description of disease-associated cortical bone loss in the clinical routine.

It is common knowledge that periarticular osteoporosis comprehend a close association to level of disease activity, but not to disease duration, even pointing at a maximal demineralisation in early RA (39-41). The pronounced relative reduction of DXR-parameters in patients with accentuated RA-activity (see Figure 2) could also confirmed in a further study which compared DXR and Quantitative Ultrasound in patients with RA (49).

In a prospective, longitudinal study of patients with early rheumatoid arthritis, Hulsmans *et al*. (50) verified a linear course of progression over the first six years and therefore recommended conventional imaging of the hands and feet every year during the first period after onset of RA. Confirming these findings, Böttcher *et al*. (30) presented a significantly decrease of DXR-BMD and DXR-MCI over a observation period of six years. In the first year of clinical RA manifestation, DXR-BMD and DXR-MCI revealed an accentuated decrease with -10.7% versus -14.3%. After a disease duration of more than one year a more flattened decline of DXR-BMD and DXR-MCI was observed. On an average this study verified an annual bone loss of -3.6% (DXR-BMD) and an annual reduction of DXR-MCI of -3.2% (30). Furthermore a second advanced longitudinal study over three years with 313 RA patients observed an annual decline of -7.4% versus -7.7% for DXR-BMD and DXR-MCI (51).

Altogether the early diagnosis of RA is essential not only for the optimal and well timed treatment of osteoporosis but also for delaying or stopping inflammatory damage of the affected joints (52). Digital X-ray Radiogrammetry seems to represent a convenient diagnostic approach for differentiating RA-related local osteoporosis.

## CONCLUSIONS

The development of digital imaging technology has promoted the precise measurement of several radiogeometric features. Digital X-ray Radiogrammetry is able to quantify disease-related demineralisation and reduction of cortical thickness dependent on severity of early RA and accentuated in patients with advanced disease activity. Possible applications and clinical importance of DXR may include BMD- and especially MCI-calculations in routinely performed follow-up radiographs for monitoring the progression of RA and for confirming reparative changes after medicamentous treatment. Therefore the operator-independent and computerized DXR technology can be an important diagnostic tool in rheumatoid arthritis by which the clinician can target those patients who require more aggressive management of their RA in order to prevent joint destruction, which will inevitably lead to disability.
